# 

**Published:** 1847-11

**Authors:** 


					﻿THE
WESTERN JOURNAL
OF
MEDICINE AND SURGERY.
EDITORS.
DANIEL DRAKE, M. D.,
PROFESSOR OF PATHOLOGY AND THE PRACTICE OF MEDICINE
IN THE UNIVERSITY OF LOUISVILLE.
LUNSFORD P. YANDELL, M. D.,
PROFESSOR OF CHEMISTRY AND PHARMACY IN THE SAME.
THOMAS W. COLESCOTT, M. D.
TO OUR SUBSCRIBERS.—Another half year is gone, and a large sum is
still due us for this Journal. We hope the delinquent subscribers will listen to
the voice of conscience within them, and remit without delay.
Sept. 1847.	PRENTICE & WEISSINGER.
The Western Journal of Medicine and Surgery is published monthly bj
the undersigned, at the corner of Main and Fifth streets, Louisville, at $5 pei
annum, payable in advance. Each number contains 96 pages, making two vol
ames in the year of more than 550 pages each.
Contributions to its pages by the Physicians of the Valley of the Mississippi
addressed to the Editors, are respectfully solicited.
Letters on business, to be addressed, postage paid, to the publishers.
January, 1844.	PRENTICE & WEISSINGER.
				

